# Wound Shock

**Published:** 1919-04-26

**Authors:** 


### WOUND SHOCK.

### Lessons Derived from the War.

In spite of the multitude of instances of this condition that the war has provided, the facts one can glean from current literature and the discussions heard at the recent B.M.A. meeting are disappointing, in that we do not seem to have arrived at a position at all comparable with the finality obtained in many other medical situations thrust upon us by the urgency of hostilities. Nevertheless, considerable advances have been made in the last two years, and some of these are well worthy of notice as leading in the right direction.

In 1914 the custom was to employ, both as preventives and as remedies, rest, artificial heat, liquid by the mouth, and saline infusion, and in the main these methods still hold the field. As the war progressed improvement in splinting and surgical technique did much to reduce the incidence of the more severe forms of shock, and therefore the older measures became relatively more adequate; but in 1917, due to the initiative of our American allies, the direct transfusion of blood came to be more extensively used, and also the gum-arabic solutions of Professor Bayliss.

Opinions vary as to which of the methods should be given preference, and it seems, on theoretical grounds, surprising that the former is not greatly and obviously superior; but, as will be seen later, there were some speakers at the meeting who still believe in the latter. Amongst surgeons at the C.C.S.S. and Base Hospitals in France opinion is unanimous in favour of blood transfusion.

It is generally recognised that in wound shock there is considerable diminution of total amount of fluid in the circulation, and there seems no doubt that one essential consists of supplying this fluid, if only by the mouth. At the B.M.A. meeting Sir Anthony Bowlby drew attention to a growing tendency to give fluids in every way other than this, and certainly, for mild cases and early stages of shock, there is much to be said for the natural route. In severe instances, however, we must of necessity employ more active measures, and of these the readers of the opening papers at South Kensington gave us first-hand, if inconclusive, evidence.

In introducing the subject Professor Bayliss said that the main factor in wound shock is a deficiency of blood in the circulation, usually, though not always, showing itself by a low blood-pressure. The fluid in circulation must be increased and a better supply of oxygen to the tissues ensured. This is the object of intravenous injection. Saline solutions in this connection have little value, and the only fluids between which the choice lay are blood and gum saline. Both are capable of increasing the volume of the blood in circulation, anl it is only reasonable to suppose that the natural fluid to replace blood is blood itself. The speaker then entered into an inquiry as to how far gum saline was capable of taking the place of blood. He thought it remarkable how little positive evidence there is on the whole as to the superiority of blood transfusion. Cases of prolonged and serious shock fail to respond both to gum saline and to blood. Unless tested adequately as regards their reaction to both, it is clear that such cases offered no evidence as to the relative want of value of gum saline. As to the cause of failure of both blood and gum in the state that results from prolonged anæmia of the tissues, there are definitely known to be two serious results, either of which renders it impossible to produce any permanent improvement of the circulation by intravenous injection of any kind. The first is paralysis of the vital nerve centres, especially of the vasomotor centre, and the second pathological state induced by prolonged low blood-pressure in the capillaries is an increase in the permeability of the blood-vessels to colloids. His general conclusion was that, of the colloids available possessing the osmotic pressure of those in blood, gum arabic in 6 per cent. solution is the best. It has to be dissolved in 0.9 per cent. sodium chloride in order to be isotonic with the blood corpuscles. Since properly prepared gum saline is harmless, no fear need be entertained of giving too much.

Dr. H. H. Dale dealt with the different conditions to which the name "shock" has been applied, dividing the cases into primary and secondary. A weakening of the vasomotor tone—due, however, to reflex inhibition rather than to fatigue of the centre—seems likely to play a large part in the primary shock, and, as to the secondary shock, he arrived at the conception of an aseptic traumatic toxæmia as playing a large part in its genesis. Traumatic toxæmia was a factor with which a perfectly healthy organism, with its powers of resistance not otherwise impaired, can often cope successfully. Many different influences can contribute to the depression of the natural resistance and power of recuperation—cold, lack of fluids, hæmorrhage, anæsthetics, such as chloroform and ether, and probably pain and exhaustion. When shock has once appeared, and the effective blood volume has become seriously depleted, the only hope would seem to lie in restoring the volume by intravenous injection on the lines which Professor Bayliss had discussed. Often, however, it is to be feared that, when shock has already become fully established, the capillary walls will have become so injured as to be no longer capable of retaining the tone necessary for an efficient circulation, or of retaining fluids which are injected.

Dr. Cowell thought that the cold factor in shock should be emphasised. Even at the present day one still found cold operating-theatres and no means of warming the table. In the greater number of wounded the blood-pressure at first was normal.

